# Working-from-home persistently influences sleep and physical activity 2 years after the Covid-19 pandemic onset: a longitudinal sleep tracker and electronic diary-based study

**DOI:** 10.3389/fpsyg.2023.1145893

**Published:** 2023-05-05

**Authors:** Stijn A. A. Massar, Ju Lynn Ong, TeYang Lau, Ben K. L. Ng, Lit Fai Chan, Daphne Koek, Karen Cheong, Michael W. L. Chee

**Affiliations:** ^1^Centre for Sleep and Cognition, Yong Loo Lin School of Medicine, National University of Singapore, Singapore, Singapore; ^2^Health Promotion Board, Singapore, Singapore

**Keywords:** sleep, physical activity, work-from-home (WFH), hybrid work, wellbeing, wearable, Fitbit, ecological moment assessment

## Abstract

**Objective:**

Working from home (WFH) has become common place since the Covid-19 pandemic. Early studies observed population-level shifts in sleep patterns (later and longer sleep) and physical activity (reduced PA), during home confinement. Other studies found these changes to depend on the proportion of days that individuals WFH (vs. work from office; WFO). Here, we examined the effects of WFH on sleep and activity patterns in the transition to normality during the later stages of the Covid-19 pandemic (Aug 2021–Jan 2022).

**Methods:**

Two-hundred and twenty-five working adults enrolled in a public health study were followed for 22  weeks. Sleep and activity data were collected with a consumer fitness tracker (Fitbit Versa 2). Over three 2-week periods (Phase 1/week 1–2: August 16–29, 2021; Phase 2/week 11–12: October 25–November 7, 2021; Phase 3/week 21–22: January 3–16, 2022), participants provided daily Fitbit sleep and activity records. Additionally, they completed daily phone-based ecological momentary assessment (EMA), providing ratings of sleep quality, wellbeing (mood, stress, motivation), and information on daily work arrangements (WFH, WFO, no work). Work arrangement data were used to examine the effects of WFH vs. WFO on sleep, activity, and wellbeing.

**Results:**

The proportion of WFH vs. WFO days fluctuated over the three measurement periods, mirroring evolving Covid restrictions. Across all three measurement periods WFH days were robustly associated with later bedtimes (+14.7 min), later wake times (+42.3 min), and longer Total Sleep Time (+20.2 min), compared to WFO days. Sleep efficiency was not affected. WFH was further associated with lower daily step count than WFO (−2,471 steps/day). WFH was associated with higher wellbeing ratings compared to WFO for those participants who had no children. However, for participants with children, these differences were not present.

**Conclusion:**

Pandemic-initiated changes in sleep and physical activity were sustained during the later stage of the pandemic. These changes could have longer term effects, and conscious effort is encouraged to harness the benefits (i.e., longer sleep), and mitigate the pitfalls (i.e., less physical activity). These findings are relevant for public health as hybrid WHF is likely to persist in a post-pandemic world.

## 1. Introduction

The global COVID-19 pandemic massively disrupted daily schedules. Lockdowns instituted to control the spread of infection confined millions of people to their homes, drastically restricting movement and social interaction. Early studies reported that the ensuing changes in routines were accompanied by marked changes in sleep, wellbeing, and physical activity. Studies in various populations across the world, have observed a shift to later sleep timing and longer sleep duration, during initial lockdowns (Blume et al., 2020; Leone et al., 2020; Wright et al., 2020; Lim et al., 2021; Ong et al., 2021b). This likely reflects that time recovered from routine activities, such as commuting, and less rigid work/school schedules, allowed individuals to more flexibly schedule their sleep according to their circadian preferences (Korman et al., 2020). On the other hand, survey studies have reported consistent worsening of subjective sleep quality and increased insomnia symptoms during the early pandemic (Amicucci et al., 2021; Cellini et al., 2021). These changes were often associated with the stress of home confinement, fear for infection, and financial hardship (Franceschini et al., 2020; Morin et al., 2020). Unsurprisingly, movement restrictions were also associated with significant reductions in physical activity (Giuntella et al., 2021; Ong et al., 2021b).

One factor that seemed directly related to these changes in sleep and activity, was whether respondents were able to work from home (WFH) or continued work in-person. A study conducted among U.S. healthcare workers in early 2020, showed that those workers who could work from home reported later and longer sleep, less physical activity, and better mood, compared to those who were required to work in-person (Conroy et al., 2021). Similarly, a comparison of essential workers and non-essential workers in Ireland observed stronger shifts in sleep timing and duration in non-essential workers, who were able to work from home (Raman and Coogan, 2022). Time-use surveys among U.S. and Swedish workers showed that WFH was associated with increased time spent in bed, and reduced travel time (Hallman et al., 2021; Restrepo and Zeballos, 2022). Furthermore, a study among Singaporean university students and staff found that, upon lifting of the lockdown, participants who continued to study or work from home more, also showed sustained patterns of longer and later sleep and lesser physical activity, but also poorer subjective sleep quality (Massar et al., 2022).

WFH during the early pandemic has been associated with particular challenges, such as balancing work and care duties (especially during school closures), social isolation, and managing eroding boundaries between work and free time (Chirico et al., 2021; Tejero et al., 2021; Shirmohammadi et al., 2022). These conditions have often led to increased stress, anxiety and depression. In addition, a lack of physical activity and the absence of suitable working space and equipment at home, has been associated with increased physical complaints such as musculoskeletal pain (Chirico et al., 2021). On the other hand, WFH has also presented new opportunities. For some, WFH increased the flexibility with which they could complete work tasks, which could have a positive impact on productivity and motivation (Shirmohammadi et al., 2022). The net outcome of WFH on health and wellbeing likely differ depending on the worker’s circumstances (e.g., age, gender, family composition) (Casagrande et al., 2020; Varma et al., 2021; Scarpelli et al., 2022), and on work-related factors (e.g., organizational support, job security) (Kniffin et al., 2021). It is likely however, that the challenges related to working from home in the later pandemic period, were not the same as those faced during initial strict lockdowns. As restriction measures started to lift (gradually), a more hybrid form of WFH and in-person work emerged. Some pressures, such as home schooling, may be relieved in later periods, and employees and organisations may have developed better modes of working as they adapted to the situation.

Whether WFH will remain as a common post-pandemic work arrangement is not fully known yet. A large-scale Gallup poll showed that, in September 2021, 45% of polled employees worked on a fully WFH schedule (25%) or on hybrid schedule combining WFH and work from office (WFO) (20%) (Saad and Wigert, 2021). While these numbers may have come down slightly, there is still lively debate about how much hybrid work should remain as a lasting part of business operations (Savage, 2021; Wingard, 2022). In some countries, policy makers have instated legislation to protect remote work beyond the pandemic (Senatori and Spinelli, 2021; Baazil and Cras, 2022).

As the pandemic evolved (and most recently devolved), new data and insights have accrued. However, most data stem from the early pandemic period. Only a few studies have examined longitudinal changes in the later pandemic period. Barbieri et al. (2021) collected fitness tracker and self-report data over a one-year period (until May 2021) in a population of US college students. Like others, they found an initial abrupt increase in sleep, reduced physical activity and an increase in mental health symptoms. As Covid-related restrictions were gradually lifted, and vaccines became more widely available, there was a gradual return to pre-pandemic sleep and mental health levels around May 2021. Physical activity levels, however, remained below pre-pandemic levels, despite a slight increase over time. Salfi et al. (2022) collected a multi-wave survey study commencing during the first lockdown in Italy (April 2020), with follow-up waves during the second lockdown (December 2020), and 2 years into the pandemic (April 2022). Similarly, they observed that many lifestyle and wellbeing outcomes gradually reverted to the pre-pandemic state (shorter and earlier sleep, lower anxiety, depression). How these changes in sleep and wellbeing relate to changes in work arrangements is not fully known. It is possible that over time people have adapted to the pandemic restriction measures or have adopted routines that allow for optimization of time use under hybrid/ WFH arrangements. As some forms of WFH/hybrid work are likely to remain in place even beyond the pandemic, it is useful to further characterize sleep, activity and wellbeing patterns as a function of work arrangements in the later pandemic period.

In the current study, we analysed data collected from a 6-month tracking study conducted in the later pandemic period (August 2021–January 2022) among Singaporean office workers (*N* = 225). During this period, Singapore experienced a protracted transition to normality. After an initial lockdown (April–June 2020), a phased reopening was instated, during which many restriction measures remained in place. As part of this, returning to in-person work was strictly regulated in Singapore for an extended period after lifting of the initial lockdown (Mathews et al., 2022). Over the course of two and a half years, the stringency of measures, including the mandated percentage of in-person vs. remote work, was dynamically adjusted to respond to changes in pandemic risk levels (e.g., fluctuating infection rates, emergence of new virus variants, and increasing vaccination rates). Relevant to the current analysis, Singapore had just experienced a period with mandatory WFH for all non-essential workers (May–August 2021). The start of our study coincided with the announcement of a gradual lifting of measures from August 2021. During this transitional period, return to in-person work became increasingly allowed (with some fluctuations due to rising case numbers). The current data were collected as part of an intervention study that was planned for this period (results reported in Ong et al., 2022). Objective sleep and activity data were collected through a consumer fitness tracker, and information on daily work arrangements and wellbeing ratings were provided through a smartphone app periodically. Our main aim for the current analysis was to examine whether the fluctuating work arrangements (WFH/WFO) in this later phase of the pandemic, would still influence living routines, or whether the earlier shifts related to WFH would have reverted to pre-pandemic levels. We additionally examined how WFH/WFO arrangements influenced wellbeing outcomes.

## 2. Methods

### 2.1. Participants and procedures

Data used in this analysis were collected as part of an intervention sub-study of the “Health Insights Singapore” (hiSG) study, a longitudinal population-health study among 1,951 working adults, initiated by the Health Promotion Board, Singapore in 2019 (Ong et al., 2021b). Two-hundred and twenty-five participants who were enrolled in the hiSG study (143 males, mean age = 35.5 ± 4.4y), were recruited to take part in a 22-week intervention study aimed at improving sleep through semi-personalised sleep goals and small incentives. The study ran during the late pandemic period from August 2021 to January 2022, overlapping with fluctuating pandemic restriction measures. Throughout the study period, participants logged their sleep and physical activity levels via a consumer fitness tracker (Fitbit Versa 2, Fitbit Inc., San Francisco, CA, United States). During three main measurement periods (Phase 1/week 1–2: August 16–29, 2021; Phase 2/week 11–12: October 25–November 7, 2021; Phase 3/week 21–22: January 3–16, 2022), they further provided daily wellbeing ratings and logs of their work arrangements (WFH, WFO, No work) via an ecological momentary assessment phone application (EMA). These records allowed us to evaluate how work arrangements changed over the main measurement periods (i.e., the proportion of WFH vs. WFO), and how this impacted sleep, physical activity, and wellbeing outcomes. It should be noted that these data were collected in the context of an intervention aimed at improving sleep (increasing sleep duration), reported in a separate communication (Ong et al., 2022)^1^. As per the aims of the intervention, participants were invited if they showed habitual short sleep (mean < 7 h/night) in a pre-study baseline period. Furthermore, participants who worked nightshifts, or were pregnant/breastfeeding mothers were excluded. Throughout the study, participants were rewarded cash-convertible credits (HealthPoints) for providing sleep, activity, and EMA data. Importantly, as the intervention did not yield significant effects during the main measurement periods, intervention and control group participants were combined for purposes of this study which focuses on the effects of work arrangements found previously to impact sleep and activity outcomes (Massar et al., 2022). All procedures were approved by the National Healthcare Group Domain Specific Review Board (DSRB), and participants signed informed consent prior to enrolling in the study.

### 2.2. Sleep and activity tracking

For sleep measures, only nights with a single nocturnal sleep period, logged by the tracker, were considered. Days with multiple separate sleep episodes (split sleep) in a day (5.7% of records), and sleep sessions <3 h were excluded. Following our prior work (Ong et al., 2021b), data was also filtered to remove days with wear time < 8 h/day or with atypical activity levels [i.e., records with (1) total daily steps >50,000, (2) no resting heart rate, or (3) no recorded steps]. Bedtime, Wake time, Time in Bed (TIB), Total Sleep Time (TST), Wake after sleep onset (WASO), and sleep efficiency (SE) were extracted as the sleep metrics of interest. Daily steps count was used as an indication of physical activity.

### 2.3. Ecological momentary assessment

During the three 2w-measurement periods, phone-based questionnaires were distributed through the hiSG phone application twice daily (Morning EMA: 8 a.m.–12 p.m., and Evening EMA: 8 p.m.–12 a.m.; see Supplementary material for description of EMA questions). Morning EMA sessions probed previous night’s sleep quality (1 – Very Poor to 5 – Very Good), present mood (Visual analogue scale: 0 – Negative to 100 – Positive), and present motivation, stress and sleepiness levels (Visual analogue scale: 0 – Not at all to 100 – Extremely). Evening EMA similarly probed present mood and stress levels, as well as work location (Home/Office/Other/No Work) for that day, and commuting time if work was done outside the home. Work from office/other location were further combined into a single category (WFO) for analysis purposes.

### 2.4. Statistical analysis

While most participants contributed more data, we set a minimum cut-off of at least 3 days per study phase with concurrent sleep recordings (Fitbit-derived) and work location information (EMA-derived). Prior analysis of the data from the main cohort indicated that, with a minimum of 3 days of Fitbit sleep data, good reliability could be reached for estimates of average sleep duration (TST, TIB) and sleep timing metrics (Bedtime, Wake time) (Lau et al., 2022). Using this criterion, 213 out of 225 participants recruited (94%) contributed data to at least 1 main measurement period [Phase 1: 207 (92%), Phase 2: 179 (80%), and Phase 3: 174 (77%)]. Included participants contributed an average (SD) of 9.72 (3.31) days in Phase 1, 9.33 (3.43) in phase 2, and 8.81 (3.55) days in Phase 3. Statistical analyses were conducted in R 4.1.0 (R Foundation for Statistical Computing, 2023). Fully unconditional null models with no predictors were first estimated, which are analogous to conducting random effects ANOVA models. From these, intra-class correlation (ICC) values representing the ratio of the between-subjects variance to the total variance were found to be substantial (0.25–0.95), indicating large between-subject differences in the sample. Thus, linear mixed-effects models were used to evaluate the effect of work location and study phase on sleep (bedtime, wake time, TIB, sleep quality) and wellbeing (morning/evening mood, morning/evening stress, sleepiness and motivation) outcome measures. Work location (WFH, WFO, No Work) and study phase (1–3) were modelled as fixed effects, while participant ID was modelled as a random effect to account for correlations across repeated observations in the same subject. Pairwise comparisons were used to test for significant differences between/within work locations and study phases.

## 3. Results

### 3.1. Descriptive statistics

A total of 213 participants were included in this analysis (males *N* = 134, age range: 26–44y). Sample descriptive information is displayed in Table 1. About 70% of participants were single or married without children, while the remaining 30% were married with children (or divorced with children). Most participants had a Bachelor’s degree or above, and worked at professional or management level (>85%). The sectors that were most represented were Finance/Insurance, Information and Communications, Education, and Manufacturing/Construction. Fitbit derived sleep and physical activity metrics (Table 2) showed no significant changes in average sleep duration and timing, as well as step counts over the different study phases (*p*’s > 0.30).

**Table 1:** Demographics and descriptive statistics of study sample.

	*N*/mean	%/stdev.
Gender (females)	79	37.09
Age (y)	35.64	4.39
*Ethnicity*		
Chinese	203	95.31
Malay	4	1.88
Indian	4	1.88
Others	2	0.94
*Family status*		
Single	116	54.46
Married w/o children	36	16.90
Married w children	56	26.29
Divorced w children	5	2.35
*Education*		
Postgraduate degree	40	18.78
Bachelor’s degree	142	66.67
Polytechnic/other professional qualifications	25	11.74
High school	6	2.82
*Sector*		
Financial/insurance	42	19.72
Information and communications	31	14.55
Education	23	10.80
Construction/manufacturing	22	10.33
Public administration and defence	19	8.92
Professional, scientific and technical services	16	7.51
Health	15	7.04
Real estate	9	4.23
Transportation, logistics, storage	7	3.29
Administrative and support services	6	2.82
Others	22	10.33
*Level*		
Senior management (director level and above)	5	2.35
Middle management (head of department/unit)	29	13.62
Professional	148	69.48
Associate professionals/technician	13	6.10
Clerical support	8	3.76
Service/sales workers	5	2.35
Plant and machine operators/assemblers	1	0.47
Not declared	3	1.41

**Table 2:** Means (sem) of Fitbit sleep and physical activity outcomes over the study phases.

	Phase 1 (Aug 2021)	Phase 2 (Oct 2021)	Phase 3 (Jan 2022)
Time in bed (TIB; min)	405 (68.2)	407 (60.1)	405 (70.7)
Total sleep time (TST; min)	354 (61.0)	354 (53.5)	353 (62.5)
Bedtime (hh:mm)	01:02 (01:24)	00:56 (01:26)	00:59 (01:22)
Wake time (hh:mm)	07:47 (01:30)	07:43 (01:34)	07:44 (01:34)
Wake after sleep onset (WASO; min)	52 (13)	53 (13)	53 (14)
Sleep efficiency (%)	90.9 (11.1)	91.2 (10.3)	91.0 (10.8)
Daily step count	9,017 (5140)	8,777 (5179)	9,587 (5,054)

### 3.2. Restriction measures and work arrangements across the study period

**Figure 1:**
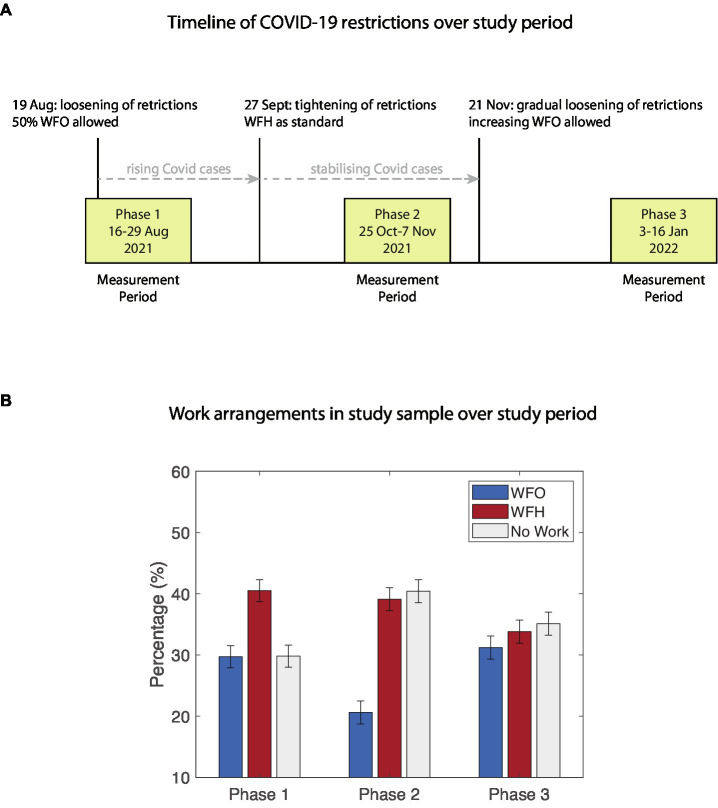
**(A)** Timeline of study events and changes in restriction policies with regards to work arrangements. **(B)** Percentages of Work From Office (WFO), Work From Home (WFH), and free days (No Work) over the study phases.

The evolving nature of the COVID-19 situation over the study period led to fluctuations in the stringency of restriction measures (see Figure 1A). During Phase 1 (16–29 Aug) several restriction measures were lifted, and businesses were allowed to have up to 50% of employees working on-site. Rising numbers of infections led to a tightening of measures, and from 27th September 2021, working from home was re-instituted as the default for employees who could do their jobs from home. From 21st November 2021, a gradual easing of measures was implemented. Accordingly, the proportion of days worked from office vs. worked from home fluctuated over the three study phases (see Figure 1B). In Phase 1 participants reported 29.7% of days WFO, 40.5% of days WFH, and 29.8% No work days. In Phase 2, the percentage of WFO dropped to 20.6%, while 39.1% of days were WFH, and 40.4% were No work days. In Phase 3, WFO increased back to 31.2%, with 33.8% WFH, and 35.1% No work days. The relative increase in No work days in Phase 2 reflects the occurrence of a public holiday on November 4, on which all participants were free. On days on which participants worked from office, average commute time was 47.7 min (SD = 21.96) in Phase 1, 45.76 min (SD = 22.03) in Phase 2, and 51.29 min (SD = 28.94) in Phase 3.

### 3.3. Effects of working from home on sleep and activity outcomes

**Figure 2:**
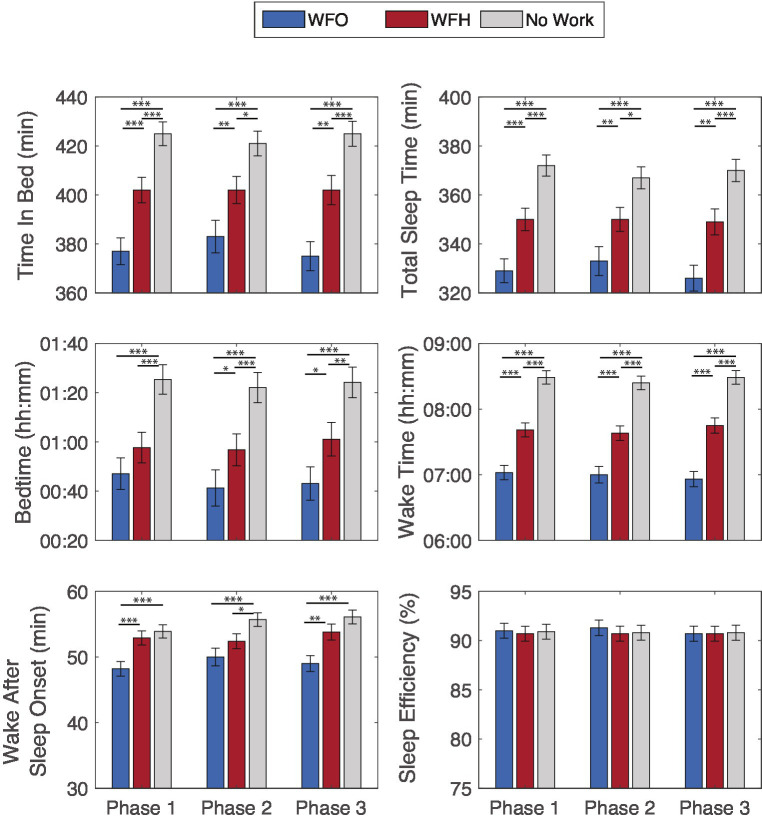
Fitbit-derived sleep outcomes by next day work arrangement, Work From Office (WFO), Work From Home (WFH), and free days (No Work) over the study phases. *p < 0.05, **p < 0.01, ***p < 0.001.

When comparing sleep periods on the prior night, significant differences between WFO, WFH, and No-work days were observed (see Figure 2). Main effects of Work arrangement were found for bedtime (*F* = 24.49, *p* < 0.001), wake time (*F* = 93.77, *p* < 0.001), TIB (*F* = 26.71, *p* < 0.001), TST (*F* = 27.36, *p* < 0.001), and WASO (*F* = 10.60, *p* < 0.001). There were, however, no significant effects of Work arrangement on sleep efficiency (*F* = 0.46, *p* = 0.63). WFH days were associated with an average (sem) of 14.7 (4.25) min later bedtime, 42.3 (5.49) min later wake time, 24 (5.14) min longer TIB, 20.2 (4.52) min longer TST, and 3.95 (0.99) min longer WASO than WFO days. On the night before No-work days bedtime was delayed by 40.1 (3.8) min, while wake time delayed by 88.1 (5.05) min, TIB increased by 45.4 (4.75) min, TST increased by 40.1 (4.18) min, and WASO increased by 6.17 (0.91) min compared to WFO days. These effects were present throughout all study phases as was evident from the absence of any phase main effects or interactions (all *p*’s > 0.30). Subjective sleep quality was better on WFH and No-Work days compared to WFO days, however, these effects were rather small (average 0.08 and 0.16 points on a 5-point scale). Similarly, morning sleepiness was significantly lower on WFH days and No-work days compared to WFO days but only by a few points on a 100-point scale (−2.30 and −3.08 points, respectively).

**Figure 3:**
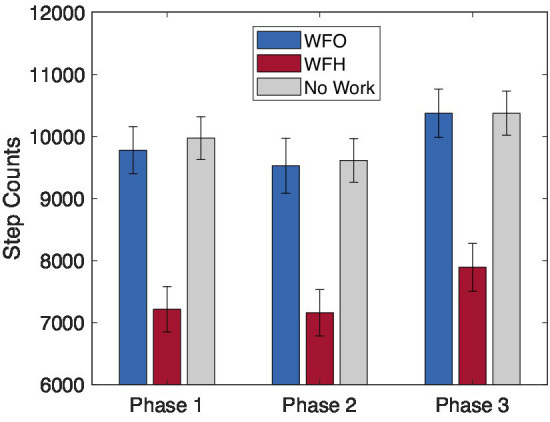
Fitbit-derived daily step count by work arrangement, Work From Office (WFO), Work From Home (WFH), and free days (No Work) over the study phases.

For daily step count, a significant main effect of Work arrangement was found (*F* = 31.00, *p* < 0.001). Paired comparisons indicated that both WFO days and No-work days were associated with significantly more daily steps (~10,000) than WFH days (~7,000) (Figure 3). This effect was present across all study phases, as was evident from the absence of a Phase main effect (*p* = 0.10) or a Work arrangement × Phase interaction (*p* = 0.96).

Since the impact of WFH was expected to be different across demographic groups, we ran a control analysis including Age, Gender, Education, and Family status (having children vs. no children) as predictors. We also included intervention group (Control vs. Intervention) as a factor, in case this affected the impact of work arrangements on sleep, activity and wellbeing outcomes. For each demographic/group variable a main effects and its interaction with work arrangement were included in the models. For all objective sleep and activity metrics, the main effect of Work arrangements remained significant (all *F*’s > 8.26, *p*’s < 0.0003). However, the effects of work arrangement were not significantly altered by inclusion of demographics in the models (interaction effects, *p*’s > 0.06). In addition, no significant main effects or interactions of intervention group were found, except for Wake time (Work Arrangement × Group effect, *p* = 0.04). However post-hoc comparisons showed very similar patterns of earlier wake time for WFO compared to WFH, and later wake times for No-Work days, for both the intervention and control groups (see Supplementary material for details).

### 3.4. Effects of working from home on wellbeing

The average daily ratings for mood, stress and motivation are displayed in Table 3. All wellbeing measures were characterized by a significant effect of work arrangements. This primarily reflected better mood, lower stress and higher motivation on No-work days compared to WFO days. WFH days did not differ in wellbeing ratings compared to WFO days, except for better evening mood. However, this increase, again, was very small (+2.79 points on a 100-point scale). Significant Phase main effects for morning and evening stress, indicated that stress ratings gradually increased towards phase 3. There were no significant Work arrangement × Phase interactions (all *p*’s > 0.19).

**Table 3:** Marginal means (sem), of EMA-based sleep and wellbeing outcomes by work arrangement over the study phases.

		Phase 1 (Aug 2021)	Phase 2 (Oct 2021)	Phase 3 (Jan 2022)
Sleep quality	WFO	3.71 (0.05)	3.65 (0.05)	3.65 (0.05)
(1–5)	WFH	3.55 (0.05)	3.64 (0.05)	3.57 (0.06)
	No-work	3.53 (0.05)	3.49 (0.06)	3.51 (0.06)
Morning sleepiness	WFO	34.4 (1.77)	32.2 (2.11)	30.4 (1.90)
(0–100)	WFH	32.1 (1.69)	28.3 (1.80)	29.7 (1.89)
	No-work	30.0 (1.62)	28.4 (1.70)	29.5 (1.77)
Morning mood	WFO	67.8 (1.48)	68.7 (1.73)	65.8 (1.57)
(0–100)	WFH	68.2 (1.42)	71.2 (1.49)	67.3 (1.56)
	No-work	73.2 (1.37)	72.3 (1.42)	71.5 (1.47)
Morning stress	WFO	29.8 (1.72)	31.9 (1.99)	31.9 (1.82)
(0–100)	WFH	29.8 (1.65)	29.4 (1.74)	30.8 (1.81)
	No-work	19.8 (1.60)	21.1 (1.66)	23.7 (1.71)
Morning motivation	WFO	60.3 (1.73)	61.2 (1.98)	61.1 (1.82)
(0–100)	WFH	60.6 (1.67)	61.2 (1.75)	61.0 (1.82)
	No-work	62.5 (1.62)	63.8 (1.68)	61.4 (1.73)
Evening mood	WFO	68.6 (1.33)	67.7 (1.53)	68.1 (1.38)
(0–100)	WFH	70.5 (1.28)	72.0 (1.34)	70.2 (1.38)
	No-work	75.2 (1.22)	74.0 (1.26)	72.0 (1.27)
Evening stress	WFO	28.6 (1.63)	29.9 (1.85)	32.4 (1.68)
(0–100)	WFH	28.5 (1.57)	29.3 (1.64)	29.5 (1.68)
	No-work	21.7 (1.51)	22.8 (1.55)	25.9 (1.56)

**Figure 4:**
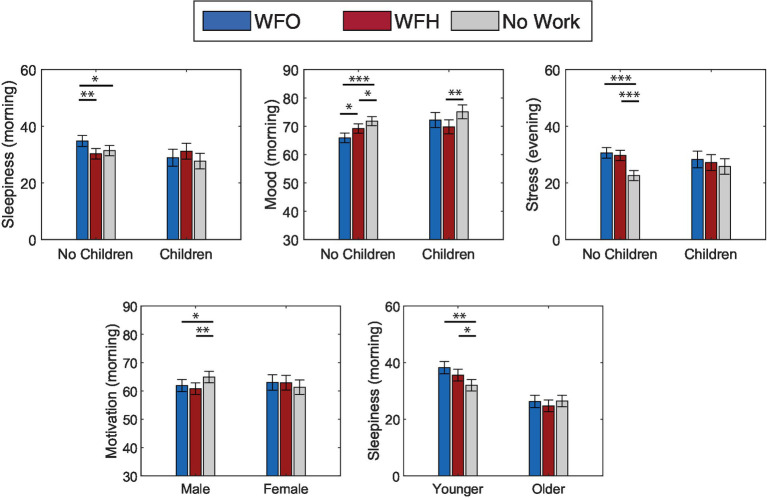
Self-reported wellbeing ratings by work arrangement, Work From Office (WFO), Work From Home (WFH), and free days (No Work) modulated by demographics *p < 0.05, **p < 0.01, ***p < 0.001.

In control analyses with demographics and intervention group as control predictors, there were significant interactions between Family status and work arrangements for morning sleepiness (*F* = 3.59, *p* = 0.03) and mood (*F* = 3.06, *p* = 0.048), as well as evening stress (*F* = 5.40, *p* = 0.005; see Figure 4). In addition, there was a significant interaction between Gender and work arrangements for morning motivation ratings (*F* = 6.30, *p* = 0.002) and between Age and work arrangements for morning sleepiness (*F* = 5.34, *p* = 0.005). Post-hoc comparisons demonstrated that participants who had no children were more sleepy and had less positive mood on WFO days compared to WFH and No Work days. For participants with children, although sleepiness and mood ratings were comparable between WFO and WFH days, poorer ratings were reported for WFH compared to No-Work days. For evening stress, participants with no children reported the lowest scores on No-Work days, compared to WFH or WFO days, while participants with children did not show any differences between days. Similarly, male participants showed the highest motivation ratings on No-Work days compared to WFH and WFO, while female participants showed no such differences. Younger participants also showed higher levels of sleepiness on WFH and WFO compared to No-Work days compared to their older counterparts.

## 4. Discussion

Evolving restriction measures over the later pandemic period (Aug 2021–Jan 2022), resulted in fluctuations in working arrangements. Working from office (WFO) was more prevalent during phases with lighter restrictions, while a larger proportion of working days were spent working from home (WFH) during periods of stricter measures. On a day-to-day basis, WFH was associated with longer and later sleep, and less physical activity. This was accompanied by small improvements in subjective sleep quality and reduced morning sleepiness during WFH days compared to WFO days. Associations between WFH and wellbeing ratings, were small, and modulated by family status, gender, and age.

These findings corroborate data from the earlier pandemic period that showed an association of WFH with more flexible sleep patterns. Across the globe later and longer sleep was observed during the initial pandemic lockdown and reopening periods (Blume et al., 2020; Cellini et al., 2020; Wright et al., 2020; Giuntella et al., 2021; Pepin et al., 2021; Rezaei and Grandner, 2021; Robbins et al., 2021; Ong et al., 2021a,b; Massar et al., 2023). The current data demonstrate that the effects of WFH on sleep behaviors did not diminish over time and were still present in January 2022. This indicates that continued WFH can persistently affect sleep and health-related routines. Throughout all study phases participants had an average of 24 min longer time in bed on WFH compared to WFO days, of which 20 min were spent asleep. Conversely, time spent commuting on WFO days averaged around 45–50 min per day. It therefore seems that a substantial portion of the time gained from not having to commute during WFH days was allocated to sleeping in later.

The effect of WFH on physical activity was equally persistent. In the early pandemic, a sharp drop in daily step count was observed upon first lockdowns (Giuntella et al., 2021; Ong et al., 2021b). Moreover, returning to WFO after the lockdown, was associated with an increase in daily steps, as compared to remaining on a WFH schedule (Massar et al., 2022). In the current data, an increase of about 3,000 daily steps during WFO days, compared to WFH days, was observed across all study phases. This indicates that, although individuals have had time and experience to adapt to new routines while working from home, the gap in physical activity between WFO and WFH days, did not narrow. It is likely that a large part of this discrepancy arose from absence of accustomed work-related activities (e.g., commuting, walking between work locations, going out for lunch).

Given the apparent persistence of the effects of WFH on sleep and physical activity, it deserves consideration as to what the long-term impact could be. With an average of about 20 min additional sleep per night, there is a higher chance for individuals to regularly obtain the recommended 7 h of sleep. Such long-term accumulation of sufficient sleep may beneficially impact health, wellbeing and cognitive performance (Pilcher and Morris, 2020). This could eventually improve work productivity and worker retention (Hillman et al., 2018). On the other hand, programs, challenges, and awareness campaigns could help to encourage physical activity when working from home (Gilson et al., 2022). The increased flexibility gained during WFH would certainly widen opportunities for scheduling exercise into the working day. As flexible work arrangements are being more broadly adopted by employers beyond the pandemic, it would be pertinent to leverage its benefits, while keeping in mind the potential pitfalls (Massar et al., 2023).

The subjective wellbeing ratings reported in this study revealed that the effects of different work arrangements were not experienced equally by all participants. WFH was associated with a reduction in sleepiness and improvement in mood, that was mostly expressed by younger participants without children. Furthermore, females and participants with children seemed to benefit less during free days, as evident from a lesser reduction in stress and lesser increase in motivation. These findings may suggest that childcare duties, and unequal gender role division of paid vs. housework may hamper the potential benefits of increased schedule flexibility when WFH.

This study has several notable strengths. Firstly, the data are based on an intensive longitudinal data collection, covering a period until early 2022, close to 2 years after the pandemic onset. While Covid restriction measures were not fully lifted by the end of the study, this time span presents a considerable outlook on the sustained impact of working arrangements on sleep, wellbeing, and activity. While a tremendous amount of information has been gathered during the initial stages of the pandemic, data on long-term effects are only starting to emerge. In addition, the current study was based on combined methods, including tracker-based measurements and phone-based self-report ratings. This allowed us to gather detailed objective data on sleep and activity (unaffected by recall bias), while still gaining insights into participants’ subjective experiences. Furthermore, the day-to-day recording of work arrangements allowed us to examine within-subject changes over a period with fluctuating restrictions.

Several limitations should also be noted. First, the current findings were part of secondary analyses of an intervention study. Delineating the effects of hybrid work was not the primary aim of the study, and information on work arrangements was collected as a precaution. Given that previous studies have observed WFH effects on sleep and activity patterns (Conroy et al., 2021; Massar et al., 2022), it could be expected that any such effect could interfere with the intervention as planned. At the start of the study, it was impossible to know, however, how the pandemic situation would play out. Consequently, it was not known how WFH/WFO arrangements would fluctuate with the evolving restriction measures. Neither was it known how strong the effects of WFH on sleep and related routines would remain in the later stages of the pandemic. As is the case with many pandemic studies, the current analysis reflects an observation of the ensuing pandemic situation, precluding the formulation of strong *a priori* hypotheses.

Relatedly, several study parameters were not specifically optimized for the current analysis. Sample size calculations were based on expected intervention effect sizes, and not on work arrangement effects. Furthermore, the sample selection may have been biased to a certain profile. Participants were pre-selected for habitual short sleep and expressed interest in improving their sleep. The resulting study sample had relatively more males than females and had a larger proportion of participants who were younger singles or married without children. It is possible that differences based on gender, family status, and age would have been more pronounced if a more balanced sample was included.

The main limitation related to this issue, howevewr, is that it is likely that participants have altered (or tried to alter) their sleep habits due to the intervention. The study evaluated the effectiveness of incentives-based nudges on improving sleep in a group of office workers (Ong et al., 2022). In the first 3 weeks of the intervention, differences between the intervention group and the control group were observed. However, these changes had fully reverted to baseline by the end of the intervention and follow-up periods (i.e., the periods included in the present analysis). Furthermore, when controlling for group membership (intervention vs. control group), the effects of work arrangements were still strongly present, and were hardly impacted by intervention treatment. Despite this lack of intervention effects, the current findings should be conservatively interpreted, and confirmation of the effects in independent studies with different study populations would be recommended.

Besides these limitations, future studies could look into optimizing the survey design, e.g., EMA timing and item construction (Ram et al., 2017; Wrzus and Neubauer, 2023), and could further examine differences between objective and subjective sleep metrics (Aili et al., 2017; Massar et al., 2021). Furthermore, the study sample could be expanded to a wider range of occupations, e.g., including healthcare workers (Conroy et al., 2021; Scarpelli et al., 2022). The modest associations between work arrangements and wellbeing found here, might also be more pronounced in those holding less secure jobs, or those whose jobs do not easily allow them to work from home (Vieira et al., 2021).

## 5. Conclusion

The current findings show that work arrangements (WFO/WFH) have persistent effects on sleep timing and duration as well as physical activity levels of working adults. As remote working arrangements are likely to remain part of post-pandemic work, it would be pertinent to devise programs to leverage the potential health benefits (e.g. reallocating to time to healthy behaviors), while mitigating the potential drawbacks (e.g. reduced physical activity).

## Data availability statement

Data may be obtained by request to the Health Promotion Board Singapore and are not publicly available. Requests to access the datasets should be directed to https://hpb.gov.sg/.

## Ethics statement

This study involving human participants was reviewed and approved by The National Healthcare Group Domain Specific Review Board. The participants provided their written informed consent to participate in this study.

## Author contributions

SM and JO have written the manuscript and have analysed the data. TL has analyzed the data. BN, LC, DK, and KC have coordinated the study. MC has provided supervision and has reviewed the manuscript. All authors contributed to the article and approved the submitted version.

## Funding

The hiSG study was developed and supported by the Health Promotion Board, Singapore. Personnel for the data analysis were additionally supported by grants from the National Medical Research Council, Singapore (NMRC/STaR/015/2013 and STaR19May-001), funds for the Centre for Sleep and Cognition, Yong Loo Lin School of Medicine, and the Lee Foundation awarded to Prof. Michael Chee.

## Conflict of interest

The authors declare that the research was conducted in the absence of any commercial or financial relationships that could be construed as a potential conflict of interest.

## Publisher’s note

All claims expressed in this article are solely those of the authors and do not necessarily represent those of their affiliated organizations, or those of the publisher, the editors and the reviewers. Any product that may be evaluated in this article, or claim that may be made by its manufacturer, is not guaranteed or endorsed by the publisher.

## Footnotes

^1^In short, an intervention group was encouraged, via small incentives, to meet two sleep goals during the 10-week intervention (sleep before midnight and sleep 30 min longer than their baseline sleep duration). A control group was compensated for just logging their sleep. While initial changes in sleep were found, sleep patterns reverted to baseline levels after 2–3 weeks into the intervention. During the main measurement periods, there were no significant differences between the intervention and control groups.
