# Supplementary material for: Working-from-home persistently influences sleep and physical activity 2 years after the Covid-19 pandemic onset: a longitudinal sleep tracker and electronic diary-based study
Source: Front Psychol. 2023 May 5;14:1145893. doi: 10.3389/fpsyg.2023.1145893 (PMC10196619; doi:10.3389/fpsyg.2023.1145893)
Supplement: Supplementary file 1 [file Presentation_1.PDF]

Supplementary Materials

Working-from-Home persistently Influences Sleep and Physical Activity 2 Years After the Covid-19 Pandemic onset: A longitudinal sleep tracker and electronic diary-based study.

Stijn A.A. Massar, Ju Lynn Ong, TeYang Lau, Ben K.L. Ng, Lit Fai Chan, Daphne Koek, Karen Cheong, Michael W.L. Chee

Daily Ecological Momentary Assessment Questionnaires

Questions to be completed in the morning (8am-12pm)

- What time did you go to sleep last night? [hh:mm]
- What time did you wake up this morning? [hh:mm]
- How was your sleep last night? [Very good, good, fair, poor, very poor]
- How are you feeling right now? [Slider – 0 (negative) -100 (positive)]
- How sleepy are you feeling right now? [Slider – 0 (not at all) -100 (extremely)]
- How motivated are you feeling right now? [Slider – 0 (not at all) -100 (extremely)]
- How stressed are you feeling right now? [Slider – 0 (not at all) -100 (extremely)]

Questions to be completed in the evening (8pm-12am)

- How are you feeling right now? [Slider – 0 (negative) -100 (positive)]
- How stressed are you feeling right now? [Slider – 0 (not at all) -100 (extremely)]
- Where did you work today? (home/office/other/did not work today)
- If you worked at the office/other location, how long did it take for you to commute from home to the office/other location? [mins/NA]

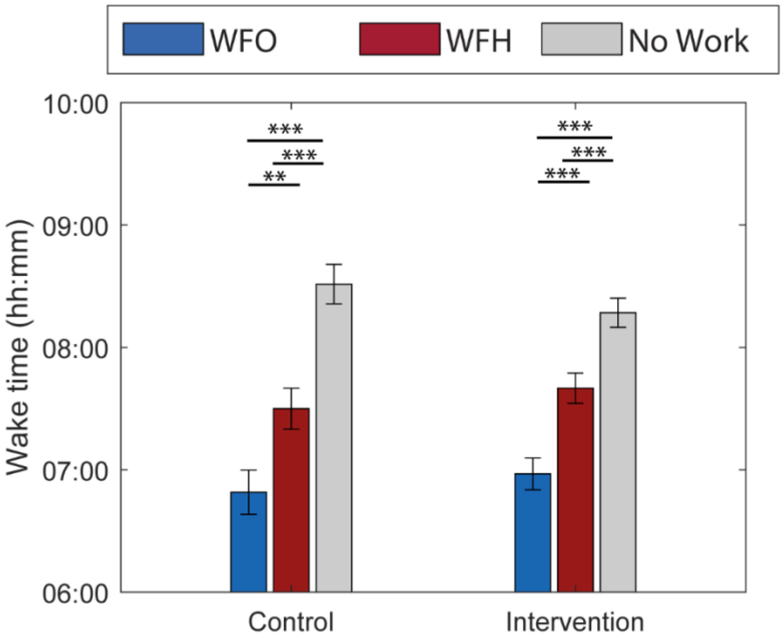

Figure S1. The effects of work arrangements on wake time separated by intervention group (control, intervention). ** p = .001, *** p < .001
